# In Memorium

**Published:** 1891-07

**Authors:** 


### In Memorium.

The following resolution by Dr. Allport in regard to the death of Dr. White, was passed at a meeting of the Chicago Dental Society held Tuesday evening, June 2d, 1891.

*Whereas:* It hath pleased the Creator and final disposer of all things to remove from this world Dr. James W. White, of Philadelphia, and

*Whereas:* It is fitting that this society should make some record of its appreciation of his virtues and of his useful life, therefore,

*Resolved:* That in the death of Dr. White, dental journalism has lost its ablest editor, the business world a member of sterling integrity, the unfortunate and needy a practical philanthropist and the church an exemplar of the nobility of a liberal christian religion.

*Resolved:* That in their affliction we extend to his bereaved family our sincere sympathy, and with reverent humility we commend them to Him who has promised to be "the friend of the widow and the fatherless" and "a real present help in the time of trouble."

*Resolved:* That a copy of these resolutions be transmitted to the family of the deceased and sent to the *Dental Cosmos, The Dental Review* and other dental journals for publication.

THE annual meeting of the Post-Graduate Dental Association of the United States was held at the Leland Hotel, Chicago, June 24, Dr. Cushing, President, in the chair.

The order of the day was reports of officers, transaction of routine business, election of officers and interesting discussions in regard to the future work of the society.

Officers elected for the ensuing year are: President, Dr. R. B. Tuller, Chicago; Vice-Presidents, Dr. Levi S. Keagle, Vinton, Ia., Dr. A. P. Nicholson, Edgerton, Wis., and Dr. M. R. Julian, Lafayette, Ind; Secretary and treasurer, Dr. L. S. Tenney, Chicago.

This organization has just completed the second year of its existence and seems to have struck a popular chord in the profession as its rapidly increasing membership would indicate.

As is generally well known the object of the society is mainly to establish a systematic course of home study and measures are now on foot to begin this work during the year.

Those desiring further information should address the Secretary, Dr. L. S. Tenney, 96 State street, Chicago, Ill.

